# Matrix Metalloproteinase-2 Polymorphisms in Chronic Heart Failure: Relationship with Susceptibility and Long-Term Survival

**DOI:** 10.1371/journal.pone.0161666

**Published:** 2016-08-23

**Authors:** Ana Rubia C. Beber, Evelise R. Polina, Andréia Biolo, Bruna L. Santos, Daiane C. Gomes, Vanessa L. La Porta, Virgílio Olsen, Nadine Clausell, Luis E. Rohde, Kátia G. Santos

**Affiliations:** 1 Laboratory of Human Molecular Genetics, Universidade Luterana do Brasil, Canoas, RS, Brazil; 2 Experimental and Molecular Cardiovascular Laboratory and the Heart Failure and Cardiac Transplant Unit, Cardiology Division, Hospital de Clínicas de Porto Alegre, Porto Alegre, RS, Brazil; Centro Cardiologico Monzino, ITALY

## Abstract

Circulating levels of matrix metalloproteinase-2 (MMP-2) predict mortality and hospital admission in heart failure (HF) patients. However, the role of *MMP-2* gene polymorphisms in the susceptibility and prognosis of HF remains elusive. In this study, 308 HF outpatients (216 Caucasian- and 92 African-Brazilians) and 333 healthy subjects (256 Caucasian- and 77 African-Brazilians) were genotyped for the -1575G>A (rs243866), -1059G>A (rs17859821), and -790G>T (rs243864) polymorphisms in the *MMP-2* gene. Polymorphisms were analyzed individually and in combination (haplotype), and positive associations were adjusted for clinical covariates. Although allele frequencies were similar in HF patients and controls in both ethnic groups, homozygotes for the minor alleles were not found among African-Brazilian patients. After a median follow-up of 5.3 years, 124 patients (40.3%) died (54.8% of them for HF). In Caucasian-Brazilians, the TT genotype of the -790G>T polymorphism was associated with a decreased risk of HF-related death as compared with GT genotype (hazard ratio [HR] = 0.512, 95% confidence interval [CI] 0.285–0.920). However, this association was lost after adjusting for clinical covariates (HR = 0.703, 95% CI 0.365–1.353). Haplotype analysis revealed similar findings, as patients homozygous for the -1575G/-1059G/-790T haplotype had a lower rate of HF-related death than those with any other haplotype combination (12.9% versus 28.5%, respectively; P = 0.010). Again, this association did not remain after adjusting for clinical covariates (HR = 0.521, 95% CI 0.248–1.093). Our study does not exclude the possibility that polymorphisms in *MMP-2* gene, particularly the -790G>T polymorphism, might be related to HF prognosis. However, due to the limitations of the study, our findings need to be confirmed in further larger studies.

## Introduction

Heart failure (HF) is a clinical syndrome of insufficient cardiac output resulting from myocardial injury that remains a leading cause of morbidity and mortality worldwide [1,2]. In addition to clinical and environmental factors, genetic variation plays a role in the development of HF, disease progression, and response to therapy [3]. Left ventricular (LV) remodeling, that precedes and occurs along the development of HF [4], is strongly associated with adverse clinical outcomes in HF patients with systolic dysfunction [5]. Changes in the overall structure and function of extracellular matrix directly contribute to the adverse LV remodeling, and evidence from animal models of LV dilation and dysfunction shows a mechanistic role for matrix metalloproteinase (MMP) proteolytic activity in this process [4,6]. In humans, circulating levels of MMPs are associated with LV remodeling, HF development, HF progression, and adverse cardiac events [7].

Matrix metalloproteinase-2 (MMP-2), also known as gelatinase A or type IV collagenase, is ubiquitously expressed in almost all cardiac cells, including cardiomyocytes. It degrades a wide range of extra- and intracellular substrates, such as denatured collagen (gelatin), collagen type IV, and sarcomeric proteins, including troponin I and myosin light chains [6,8]. MMP-2 contributes to the heart tube formation, angiogenesis [8], and early myocardial injury [8–10]. High circulating levels of MMP-2 predict LV remodeling after myocardial infarction [11,12] and are associated with hospital admission and mortality in patients with systolic HF [13–16]. Elevated MMP-2 levels are also found in stable HF patients in comparison with decompensated HF [17] and control subjects [7].

Polymorphisms in the promoter of *MMPs* genes have been associated with coronary artery disease [18], myocardial infarction [19], and HF susceptibility [20–22] and prognosis [23,24]. We previously reported that the 2G allele of the *MMP-1*–1607 1G/2G polymorphism was associated with better prognosis in patients with systolic HF [25]. We also showed that HF patients with the *MMP-1* 2G2G genotype and the 6A allele of the *MMP-3*–1171 5A/6A polymorphism had increased QRS widening rate [26]. Despite the role of MMP-2 in LV remodeling and HF prognosis, the impact of *MMP-2* polymorphisms on HF remains unexplored and inconclusive [20,22–24].

In this study, we tested the hypothesis that the -1575G>A (rs243866), -1059G>A (rs17859821), and -790G>T (rs243864) polymorphisms in the *MMP-2* gene promoter are associated with susceptibility to HF and could predict all-cause death and HF-related death in Brazilian outpatients with reduced LV ejection fraction (LVEF). We selected these polymorphisms based on intellectual choice. Functional assays and prediction analyses showed that the variants at the nucleotide positions -1575 and -790 are located near or within transcription factor binding sites, thereby affecting the levels of transcription [27,28]. The -1059G>A polymorphism, on the other hand, does not appear to be functional [28]. Nevertheless, this polymorphism was associated with HF prognosis in a Chinese population [24]. We found that HF-related death rate differed among the *MMP-2* genotypes in Caucasian-Brazilians. However, positive associations were lost in the multivariate analyses.

## Methods

### Study Population

We enrolled 308 patients with HF of any etiology and reduced LVEF (≤ 45%), diagnosed according to the ACCF/AHA guidelines [29]. Patients were recruited consecutively in the Heart Failure and Transplant Outpatient Clinic in a tertiary care university hospital (HCPA) in Porto Alegre, Brazil from July 2003 to November 2007. At baseline, patients underwent a comprehensive clinical and biochemical evaluation consisting of physical examination, assessment of electro- and echocardiographic parameters, and laboratory exams. Ischemic etiology was defined as previously reported [25]. The prognostic end points were all-cause death and HF-related death, defined as sudden unexpected death (within 1 hour of onset of symptoms) or caused by advanced refractory disease. Vital status was assessed at hospital electronic records, by telephone contact, or at the State Death Certificate Database. Survival data were last updated on April 2014.

We also enrolled a group of 333 healthy controls composed by unrelated blood donors at the Hemotherapy Center of the same hospital at the same period as HF patients. Those with a history of cardiovascular disease or related symptoms, and those with a positive family history of premature sudden death were excluded. No additional laboratory data were collected from blood donors. This study is reported according to STREGA guidelines [30]. The study protocol was approved by the Research Ethics Committee of Hospital de Clínicas de Porto Alegre (Institutional Review Board [IRB] 0000921 –application number 03–237), and was performed in accordance with the Declaration of Helsinki (Resolution 196/96 of the National Health Council). All HF patients and blood donors gave written informed consent.

As skin color and self-reported ethnicity are not reliable indicators of genetic ancestry [31], ancestry informative markers would be useful to confirm ethnicity of study participants. However, assuming that European ancestry is prevalent in Southern Brazil [32] and people defined as White have a predominant European ancestry, whereas people defined as Brown/Black have European- and African-ancestry in similar proportions [33], the ethnic classification of HF patients and blood donors was self-reported, being defined in this study as Caucasian- or African-Brazilian.

### Genotyping

Genomic DNA was isolated from peripheral blood leukocytes using a salting out procedure as described elsewhere [34], quantified, diluted to the concentration of 100 ng/μL, and stored at -20°C until genotyping, which was done at the Laboratory of Human Molecular Genetics of Universidade Luterana do Brasil. *MMP-2* genotypes were determined by polymerase chain reaction and restriction fragment length polymorphism (PCR-RFLP) method, using the primers and restriction enzymes (New England Biolabs, Ipswich, MA, USA) as previously described by Hua et al. [22]. The digested amplicons were electrophoresed on 8% polyacrylamide gels (for the -790G>T polymorphism) or on 2% agarose gels (for the variants at -1575 and -1059 nucleotide sites), stained with ethidium bromide, and visualized under ultraviolet light. To improve genotyping accuracy, samples with known genotypes were used in each run and laboratory personnel were blinded to the clinical status of the subjects. Details of the genotyping method are described in S1 Table.

### Statistical Analysis

Continuous variables with normal distribution were compared between groups by the Student t-test and those with skewed distribution were compared with the Mann-Whitney U test. The Kolmogorov-Smirnov test was used to verify the normality of quantitative variables. Categorical variables, including the genotype and allele frequencies, were compared by the Pearson chi-square or the likelihood-ratio chi-square test, as appropriate. Allele frequencies were determined by gene counting and departures from Hardy-Weinberg equilibrium were verified with the chi-square test. Pairwise linkage disequilibrium (LD) among the *MMP-2* polymorphisms was calculated and expressed in terms of D’ and r^2^ [35]. Haplotype frequencies were estimated by a Bayesian method using PHASE program (version 2.1) [36,37]. We also used PHASE to compare the distribution of different *MMP-2* haplotypes between groups of subjects, computing P-values by a permutation test with 1,000 random replicates.

To evaluate whether *MMP-2* gene polymorphisms are related to HF prognosis, Kaplan-Meier survival curves were constructed considering the period between the date of the first visit at the outpatient clinic and the last registry of follow-up or death. Survival curves obtained for the different genotypes and haplotypes were compared by the log-rank test. The association of *MMP-2* gene polymorphisms with all-cause and HF-related death was also evaluated by Cox regression analysis with estimates of hazard ratio (HR) and 95% confidence interval (CI). Whenever appropriate, models were adjusted for the demographic and clinical variables that were associated with HF prognosis in the univariate analyses.

Considering that there are interethnic differences in the distribution of *MMP-2* gene polymorphisms (1000 Genomes Project database available at http://www.ncbi.nlm.nih.gov/variation/tools/1000genomes/) and that genotype frequencies of the -1575G>A and -790G>T polymorphisms were different in Caucasian- and African-Brazilian blood donors in our study (S2 Table), all statistical analyses were stratified by self-reported ethnicity. Statistical analyses were done using SPSS (version 18.0; SPSS Inc., Chicago, IL) and WINPEPI (version 11.43) [38] statistical softwares. Power estimates were calculated with PS Power and Sample Size Calculations program (version 3.1.2) [39], and P-values < 0.05 were considered as statistically significant.

## Results

### Clinical and Demographic Characteristics

Baseline characteristics of HF patients are summarized in Table 1. Caucasian- and African-Brazilians were predominantly middle-aged male, in New York Heart Association (NYHA) class I or II, and had moderate to severe left ventricular dysfunction. Caucasian-Brazilians had predominantly ischemic etiology, whereas African-Brazilians had more often hypertensive HF (Table 1). Most blood donors were male (approximately 68% in both ethnic groups) and younger than HF patients (overall mean age 46 ± 11 years and 60 ± 13 years, respectively; P < 0.001).

**Table 1:** Baseline Characteristics of Heart Failure Patients According to Self-Reported Ethnicity.

	Caucasian-Brazilians (n = 216)	African-Brazilians (n = 92)	P-value^a^
Age (years)	60 ± 13	60 ± 12	0.711
Male, n (%)	141 (65.3)	66 (71.7)	0.331
HF etiology			
Ischemic, n (%)	90 (41.7)	24 (26.1)	**0.014**
Idiopathic, n (%)	66 (30.6)	23 (25.0)	0.397
Hypertensive, n (%)	43 (19.9)	32 (34.8)	**0.008**
History of smoking, n (%)	93 (43.1)	51 (55.4)	0.062
NYHA classes I and II, n (%)^b^	171 (81.0)	66 (72.5)	0.134
Previous myocardial infarction, n (%)	79 (36.6)	19 (20.7)	**0.009**
Electrocardiogram			
QRS duration (ms)	120 (100–154)	118 (102–153)	0.888
Echocardiography			
LV end-diastolic diameter (mm)	6.6 ± 1.0	6.6 ± 0.9	0.959
LV end-systolic diameter (mm)	5.6 ± 1.1	5.6 ± 0.9	0.896
LV ejection fraction (%)	31.4 ± 8.2	31.0 ± 8.2	0.667
Creatinine (μmol/L)	106 (80–124)	115 (88–141)	0.315
Sodium (mEq/L)	140.6 ± 3.2	140.0 ± 3.9	0.325
Hemoglobin (g/dL)	13.2 ± 1.6	13.0 ± 1.9	0.385
HF medications			
Beta-blocker, n (%)	192 (88.9)	78 (84.8)	0.416
ACE inhibitor, n (%)	186 (86.1)	84 (91.3)	0.281

HF, heart failure; NYHA, New York Heart Association; LV, left ventricular; ACE, angiotensin-converting enzyme. Data are expressed as mean ± standard deviation, median (25^th^-75^th^ percentiles) or absolute number (percentage).

^a^ P-values for the comparisons between Caucasian- and African-Brazilians were calculated using unpaired Student t, Mann-Whitney U, or χ² tests, as appropriate. Significant P-values are shown in bold.

^b^ Data available for 211 Caucasian-Brazilians and 91 African-Brazilians.

### *MMP-2* Polymorphisms and Susceptibility to Heart Failure

Genotype frequencies did not deviate significantly from those predicted by the Hardy-Weinberg equation for all polymorphisms in Caucasian-Brazilians and African-Brazilian HF patients. However, we found more homozygotes for the minor allele than the expected for the 3 polymorphisms among African-Brazilian blood donors. As shown in Table 2, the allele frequencies were similar in HF patients and blood donors for all polymorphisms in both ethnic groups. However, among African-Brazilians, genotype frequencies were different between HF patients and blood donors, as none of the HF patients was homozygote for the minor alleles.

**Table 2:** Genotype and Allele Frequencies of *Matrix Metalloproteinase-2* Gene Polymorphisms in Heart Failure Patients and Healthy Blood Donors.

		Caucasian-Brazilians			African-Brazilians		
Polymorphisms		HF Patients	Blood Donors	P-value^a^	HF Patients	Blood Donors	P-value^a^
-1575G>A, n		216	255		92	75	
Genotypes, n (%)	GG	141 (65.3)	165 (64.7)	0.307	65 (70.7)	58 (77.4)	**0.010**
	GA	67 (31.0)	86 (33.7)		27 (29.3)	13 (17.3)	
	AA	8 (3.7)	4 (1.6)		**0**	**4 (5.3)**	
Alleles, %	G	80.8	81.6	0.824	85.3	86.0	0.986
	A	19.2	18.4		14.7	14.0	
-1059G>A, n		206	235		86	74	
Genotypes, n (%)	GG	157 (76.2)	170 (72.4)	0.468	65 (75.6)	55 (74.3)	**0.016**
	GA	47 (22.8)	60 (25.5)		21 (24.4)	14 (18.9)	
	AA	2 (1.0)	5 (2.1)		**0**	**5 (6.8)**	
Alleles, %	G	87.6	85.1	0.325	87.8	83.8	0.386
	A	12.4	14.9		12.2	16.2	
-790G>T, n		216	249		89	73	
Genotypes, n (%)	GG	11 (5.1)	8 (3.2)	0.590	**0**	**4 (5.5)**	**0.010**
	GT	73 (33.8)	87 (34.9)		27 (30.3)	13 (17.8)	
	TT	132 (61.1)	154 (61.9)		62 (69.7)	56 (76.7)	
Alleles, %	G	22.0	20.7	0.685	15.2	14.4	0.968
	T	78.0	79.3		84.8	85.6	

HF, heart failure. Data are expressed as absolute number (percentage) or percentage.

^a^ P-values for the comparisons between heart failure patients and blood donors were calculated using the Pearson chi-square or the likelihood-ratio chi-square test, as appropriate. Frequencies that deviate significantly from expected in the analysis of adjusted residuals and significant P-values are shown in bold.

Seven of 8 expected haplotypes were observed in all groups of subjects, and 3 haplotypes accounted for more than 96% of the chromosomes. As shown in Table 3, haplotype frequencies in HF patients were very similar to those observed in blood donors, in both ethnic groups. Moreover, LD analysis indicated that the -1575G>A and -790G>T polymorphisms were in strong linkage in all groups of subjects (S3 Table).

**Table 3:** Haplotype Frequencies of *Matrix Metalloproteinase-2* Gene Polymorphisms in Heart Failure Patients and Healthy Blood Donors.

	Caucasian-Brazilians		African-Brazilians	
Haplotype	HF Patients	Blood Donors	HF Patients	Blood Donors
n^a^	432	504	178	146
-1575G/-1059G/-790T	0.6571	0.6469	0.7139	0.6895
-1575G/-1059G/-790G	0.0267	0.0192	0.0165	0.0001
-1575G/-1059A/-790T	0.1183	0.1443	0.1230	0.1666
-1575G/-1059A/-790G	0.0058	0.0033	0.0005	< 0.0001
-1575A/-1059G/-790T	0.0046	0.0021	0.0113	0.0001
-1575A/-1059G/-790G	0.1873	0.1841	0.1346	0.1437
-1575A/-1059A/-790T	0.0001	< 0.0001	-	-
-1575A/-1059A/-790G	0.0001	0.0001	0.0002	< 0.0001
P-value^b^		0.754		0.184

^a^ Relative frequency is based on the total number of chromosomes (instead of number of subjects).

^b^ P-values were computed by a permutation test comparing heart failure patients with healthy blood donors in the PHASE program.

### *MMP-2* Polymorphisms and Prognosis of Heart Failure

**Fig 1:**
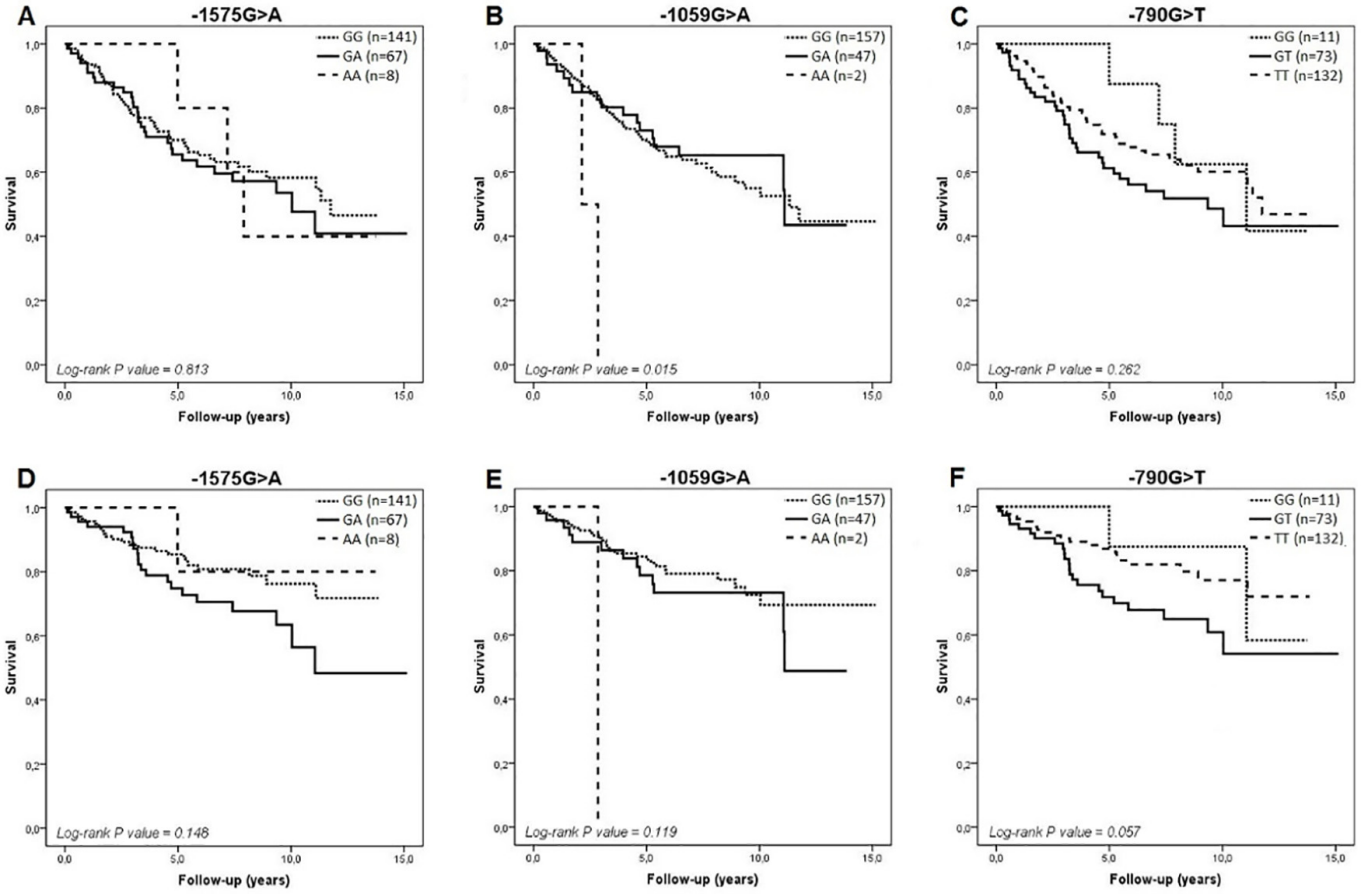
Kaplan-Meier survival curves in Caucasian-Brazilians for all-cause death (A to C) and HF-related death (D to F) according to the -1575G>A, -1059G>A, and -790G>T polymorphisms in *MMP-2* gene, respectively.

During a median follow-up of 5.3 years (25^th^-75^th^ percentiles of 2.8 and 8.3 years, respectively), 124 of 308 HF patients (40.3%) died, of whom 83 were Caucasian- and 41 were African-Brazilians. Among the 124 patients who died, 68 (54.8%) had HF-related death (47 Caucasian- and 21 African-Brazilians). Of note, 93 of the 124 deaths (75%) occurred within the first 5 years of follow-up. Fig 1 shows the Kaplan-Meier survival curves for all-cause death and HF-related death in Caucasian-Brazilians according to the -1575G>A, -1059G>A, and -790G>T polymorphisms in *MMP-2* gene, respectively, for the entire period of follow-up (maximum 15 years).

**Fig 2:**
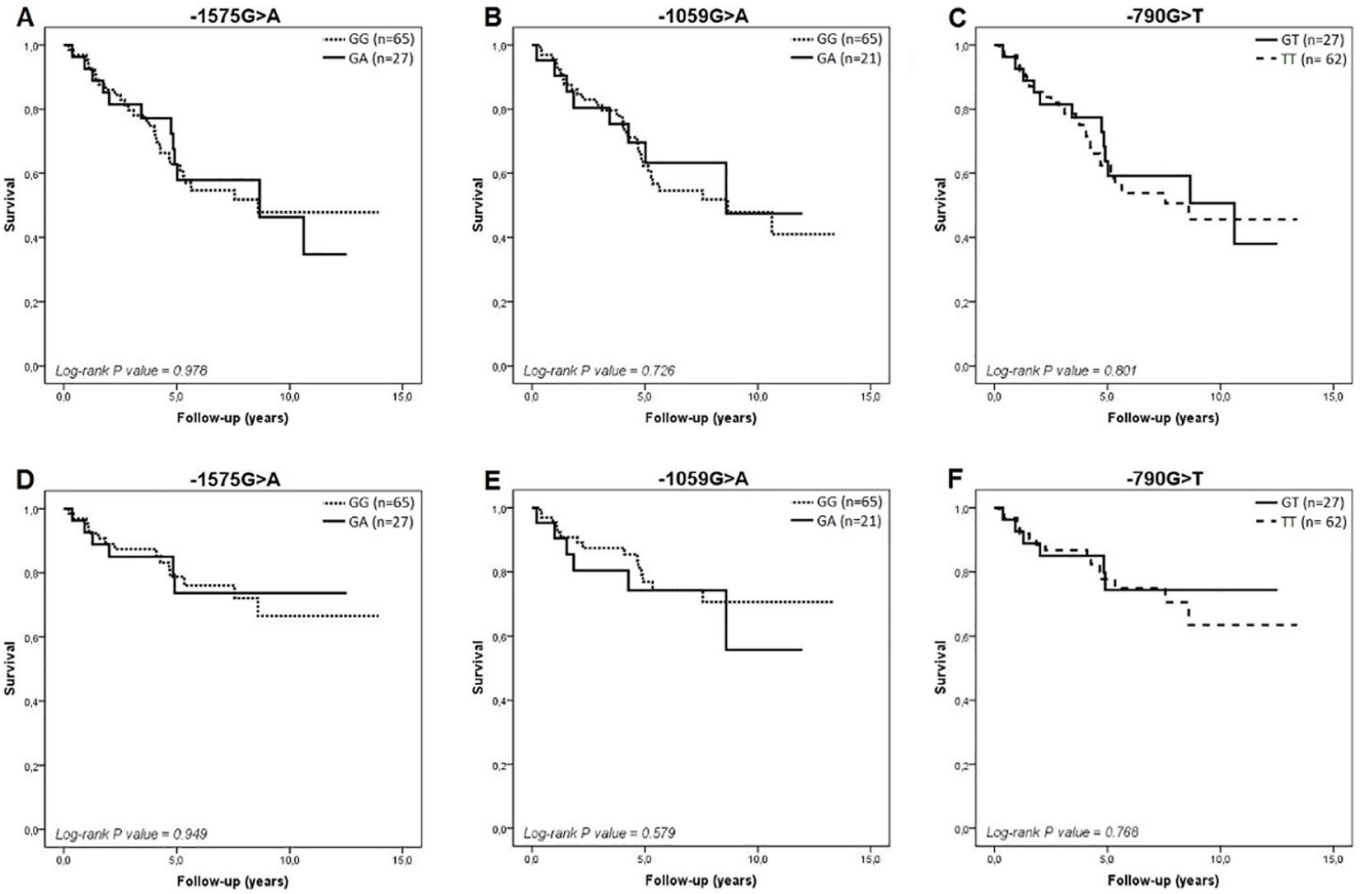
Kaplan-Meier survival curves in African-Brazilians for all-cause death (A to C) and HF-related death (D to F) according to the -1575G>A, -1059G>A, and -790G>T polymorphisms in *MMP-2* gene, respectively.

In relation to all-cause and HF-related mortality, we observed that heterozygous patients had the worst survival rates. However, except for the -1059G>A polymorphism, these differences were not statistically significant when we considered the entire period of follow-up, as indicated in the Fig 1 (log-rank P-values > 0.05). Among African-Brazilian patients, survival curves were quite similar in heterozygotes and homozygotes for the major alleles for all polymorphisms (Fig 2).

Next, we evaluated the association of the -1575G>A, -1059G>A, and -790G>T polymorphisms with HF prognosis using Cox regression analysis. The TT genotype of the -790G>T polymorphism was associated with a decreased risk of HF-related death as compared with GT genotype (HR = 0.512, 95% CI 0.285–0.920) in Caucasian-Brazilians. In fact, the rate of HF-related death was lower in TT homozygous patients than in heterozygous patients (16.7% versus 31.5%, respectively, Bonferroni-corrected P-value for pairwise comparisons between genotypes = 0.047). However, this association was lost after controlling for NYHA functional class, cigarette smoking, stroke/transient ischemic attack, systolic blood pressure, QRS duration, LV ejection fraction, LV end-systolic diameter, sodium, and hemoglobin levels (S4 Table).

**Fig 3:**
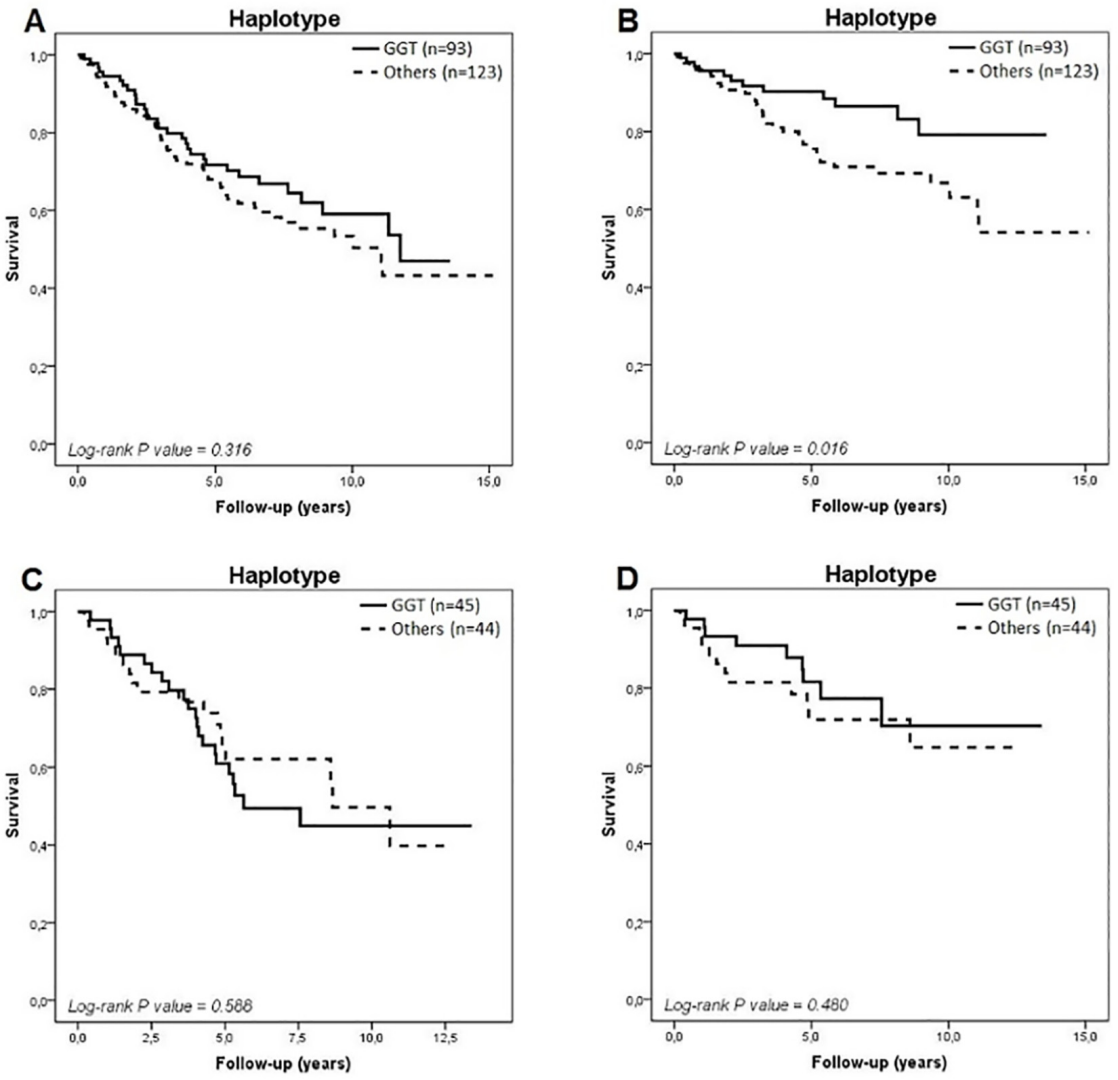
Kaplan-Meier survival curves in Caucasian- (A and B) and African-Brazilians (C and D) for all-cause death and HF-related death, respectively, according to the *MMP-2* haplotypes.

Survival analysis of *MMP-2* haplotypes (Fig 3) revealed a similar profile as that observed for individual polymorphisms, as Caucasian-Brazilians homozygous for the haplotype composed of the major alleles (-1575G/-1059G/-790T) had a better HF-related survival rate than those who carried at least 1 copy of either -1575A/-1059G/-790G, -1575G/-1059G/-790G, or -1575G/-1059A/-790T haplotypes (Fig 3B). Over the entire period of follow-up, the rate of HF-related death was 55% lower in homozygous patients for the -1575G/-1059G/-790T haplotype than in those who carried other haplotypes (12.9% versus 28.5%, respectively, P-value = 0.010). Again, the association detected between *MMP-2* haplotypes and HF-related death (HR = 0.457, 95% CI 0.237–0.880) was lost after controlling for clinical covariates (S5 Table).

## Discussion

In this study we investigated whether the -1575G>A, -1059G>A, and -790G>T polymorphisms in the *MMP-2* gene promoter, either alone or in combination, were associated with HF susceptibility and/or HF prognosis in a cohort of outpatients with reduced LVEF in an European- and African-derived population from Southern Brazil. We found that genotype frequencies of the 3 *MMP-2* polymorphisms in African-Brazilians differed in HF patients and blood donors, whereas the -790G>T polymorphism and the -1575G/-1059G/-790T haplotype were associated with HF-related death in Caucasian-Brazilians in the univariate analyses.

In Caucasian-Brazilians, the similarity in the haplotype, genotype, and allele frequencies in HF patients and healthy blood donors of the -1575G>A, -1059G>A, and -790G>T polymorphisms indicated that these gene variants are not associated with the development of HF. These findings are partly conflicting with those reported in the northern Han Chinese population from Beijing region [22], in which the -1575A allele was associated with a lower risk of systolic HF and the -1575G/-1059G/-790G haplotype was associated with a higher risk of HF. Considering that the frequencies of the -1575A allele and the -1575A allele carriers were virtually the same in HF patients and blood donors (differences < 1%), our study had a power of approximately 54%, at a significance level of 5%, to detect an unadjusted odds ratio of 0.66 for the association between the -1575A allele and HF, under the dominant model, as reported in the Chinese population [22]. We could have also failed to detect an association between the -1575G/-1059G/-790G haplotype and HF because of its very low frequency (2.2% in Caucasian- and 0.9% in African-Brazilians). Even if the -1575G/-1059G/-790G haplotype doubled the risk of HF, as suggested by Hua et al. [22], it would have a low clinical impact in our population.

In relation to African-Brazilians, the frequencies of the homozygous genotypes for the minor alleles of the 3 *MMP-2* polymorphisms were nearly 2.5-fold higher than the expected in healthy blood donors. This suggests that the carriership of the -1575AA, -1059AA, or -790GG genotypes could be associated with a reduced risk of HF, thus partly in line with the Chinese study [22]. In the report by Hua et al. [22], the genotype frequencies of the -790G>T polymorphism deviated from Hardy-Weinberg equilibrium in the control group, with an excess of homozygotes being observed, as also found in our study. Curiously, an earlier study reported that the genotype frequencies of the -1575G>A polymorphism deviated from the expected Hardy-Weinberg distribution in 2 independent study populations consisting of healthy adult blood donors and newborns of north German Caucasian origin [28].

In relation to HF prognosis, the TT genotype of the -790G>T polymorphism and the haplotype carrying all major alleles in homozygosis (-1575G/-1059G/-790T) were associated with a decreased risk of HF-related death in Caucasian-Brazilians. However, these associations were lost after adjusting for clinical covariates. These findings partially agree with those reported by Hua et al. [24] in the northern Han Chinese population, in which the carriers of either the -1059A allele or the -1575G/-1059A/-790T haplotype had a better HF prognosis (lower cardiac death rate and lower rate of a composite end point of cardiac death, readmission for HF, reinfarction, and revascularization). Our study had a power of at least 80%, at a significance level of 5%, to detect hazard ratios of ≤ 0.51 for the association of genotypes homozygous for the major alleles (-1575GG, -1059GG, and -790TT) with all-cause and HF-related death in comparison with the respective heterozygotes.

Differences in the prevalence of cardiovascular diseases, natural history of HF, and response to therapy have been attributed, among other factors, to interethnic differences in the frequency of common genetic variants [40,41]. Our findings reinforce this assumption, as we also detected several differences in the genotype and allele frequencies of the 3 *MMP-2* polymorphisms comparing Caucasian-Brazilian, African-Brazilian and north Han Chinese controls [22]. Although Caucasian- and African-Brazilian HF patients had a similar demographic and clinical profile at baseline, *MMP-2* polymorphisms seem to be associated with HF-death only in Caucasians. The absence of such an association in African-Brazilians due to lack of statistical power is unlikely, as the survival curves were virtually identical in both heterozygotes and homozygotes for the major alleles. Population differences could explain, at least in part, the contrasting findings observed by us and Hua et al. [22,24]. In line with this, Kim et al. [42] reported increased plasma levels of MMP-2 in women of African-American ethnicity as compared with women of non-Hispanic White ethnicity. Theoretical models suggest that the ‘flip-flop’ association (i.e., association of opposite alleles with the same disease) may indicate heterogeneous effects of the same variant that are due to differences in genetic background or environment [43].

Brazilian population is known to be highly admixed. However, a study using ancestry informative markers showed that people living in the four most populous Brazilian regions have a predominant European ancestry (77.7%), regardless of the color/race category. In Rio Grande do Sul State, where our study was done, people defined as White have a predominant European ancestry (85.5%), whereas people defined as Brown/Black have European- (44%) and African-ancestry (45%) [33]. One recent study with HF patients from Southeastern Brazil (São Paulo State) reported that European ancestry was associated with increased odds of having ischemic cardiomyopathy, whereas African genomic ancestry was associated with increased chance of having hypertensive cardiomyopathy [44]. Despite its limitation, our ethnic classification reflects the findings reported by Cardena et al. [44], as the ischemic etiology was more frequent among Caucasian-Brazilians, whereas the hypertensive etiology was the most frequent among African-Brazilians. Even though we did not use the ancestry informative markers to define ethnicity, stratifying the analyses by the two most prevalent self-declared ethnic groups in our region should have minimized the potential bias of population stratification.

In this context, heterogeneity of HF etiology is another factor that could be thought as a confounder. In the Chinese studies [22,24], patients had reduced LVEF caused by myocardial infarction in the past or idiopathic dilated cardiomyopathy, whereas our cohort was composed of patients who had ischemic, idiopathic, hypertensive, valvular, chagasic, or alcoholic cardiomyopathy. However, the inclusion/exclusion criteria adopted in our study are comparable to those of Hua et al. [22,24], and when we repeated all the analyses excluding the patients who had HF of valvular, chagasic, or alcoholic etiology (23 Caucasian- and 15 African-Brazilians), the results remained essentially the same (S6 Table and S1 Fig).

*MMP-2* gene polymorphisms have been investigated in association studies based on their putative effects on gene expression at the transcriptional level. However, data on the functionality of the *MMP-2* variants investigated in this study are still scarce and inconclusive, with both alleles of the -1575G>A and -790G>T polymorphisms being linked to higher and lower transcriptional activity, depending on the cell type evaluated [27,28]. The -1575G>A polymorphism is located immediately adjacent to a potential half-palindromic estrogen receptor binding site and functional characterization of this polymorphism was done by transfection experiments [27,28] and gel shift assays [28] in multiple cell lines with differential expression of alpha- and beta-estrogen receptors. It included epithelial cells (293), macrophages (RAW264.7), smooth muscle cells (A10) [27] and breast carcinoma cells (MCF-7 and MDA-MB-231) [28]. Although considered by Price et al. [27] as a neutral variant, as it did not reach the proposed minimum threshold of a 2-fold difference in allelic expression to be considered an indicator of functionality, estrogen receptor beta-negative cell lines (293 and RAW264.7) transfected with -1575A allele constructs expressed nearly 1.5-fold higher activity than -1575G transfectants. The opposite was observed in A10 cell line, that expresses estrogen receptor-beta (but not alpha), with the -1575G transfectants expressing 1.3-fold higher activity than the -1575A allele constructs. Later, Harendza et al. [28] reported that the -1575G allele functioned as an enhancer, whereas the -1575A allele reduced transcription activity (more than 50%) in constitutively estrogen receptor-positive MCF-7 cells. Additional experiments showed that these differences in allelic expression were related to the binding of the estrogen receptor-alpha to this region. Similar assays were done to evaluate the functional activity of the -1059G>A polymorphism and no differential allelic expression was detected [28].

In relation to the -790G>T polymorphism, *in silico* analysis showed that the -790 variant site maps onto a region with identical match to an inverted GATA-1 consensus sequence in the presence of the T allele, thus potentially creating a binding site for this transcription factor, which could enhance the transcription rate of the *MMP-2* gene [27]. This is in line with what was observed in the study of Price et al. [27], in which the 293 and RAW264.7 cell lines transfected with -790T allele constructs had a higher expression than the counterparts carrying the -790G allele (1.5- and -1.3-fold, respectively). In A10 cells, in contrast, the -790T allele showed a lower promoter activity compared with the -790G allele (0.9-fold). These data suggest that polymorphisms in the promoter of *MMP-2* gene might influence gene expression in a cell-type specific manner, as already discussed by Price et al. [27]. As *MMP-2* is an estrogen-responsive gene [27] and cardiomyocytes are estrogen-responsive cells, expressing estrogen receptors -alpha and -beta [45], a potential effect of the -1575G>A and -790G>T polymorphisms on *MMP-2* gene expression in the failing myocardium cannot be ruled out.

Despite the differences in the distribution of *MMP-2* polymorphisms among different populations, we observed that the -1575G>A and -790G>T polymorphisms were tightly linked in a similar magnitude in Caucasian- and African-Brazilians, as also seen among controls in the Chinese study [22]. The fact that the -1575A allele is linked to the -790G allele (while the -1575G allele is linked to the -790T allele) means that the association detected between the -790TT genotype and HF-related death in our study might reflect the effect of other variants that act in concert with the -790G>T polymorphism, thus influencing the HF prognosis. In their functional study, Price et al. [27] observed that the effect of the -790G>T polymorphism on allelic expression also varied according to which allele was present at nucleotide position -787. Moreover, Harendza et al. [28] reported that the -1575G>A and -1306C>T polymorphisms were in complete LD, and functional experiments showed an additive reduction in estrogen-dependent reporter activity of the linked variant alleles compared with the wild-type alleles. Taken together, these data indicate that the effect of individual polymorphisms on *MMP-2* gene expression is modest and might influence the susceptibility and prognosis of HF in an additive manner. Considering there are interpopulation differences in the allele frequencies of *MMP-2* variants, their functional effect may vary according to genetic background, thus leading to differential findings across populations.

Given the evidence from observational studies in humans demonstrating that MMP-2 levels are positively correlated with LV remodeling early after myocardial infarction and worse HF prognosis [11–17], we would expect that alleles that are less transcriptionally active were associated with decreased risk of developing HF and death. On the other hand, estradiol and its receptors exert protective effects on cardiomyocytes, whereby they regulate cardiac metabolism, attenuate cardiomyocyte apoptosis, promote cardiac regeneration, modulate physiological and pathological LV hypertrophy, and calibrate electrical and contractile function of the heart [45]. In this scenario, allelic variants of the *MMP-2* gene with reduced transcriptional activity would promote a diminished response to estradiol, thus reducing its beneficial effects on cardiac function, especially in HF patients (who already have structural and functional LV impairment). This could explain the seemingly paradoxical findings from previous studies that showed that patients carrying genotypes associated with higher expression of *MMPs* had a more favourable HF prognosis [25], and reduced LV mass index and LV end-diastolic diameter [46]. Overall, our data suggest that the TT genotype of the -790G>T polymorphism, putatively associated with increased *MMP-2* gene expression, might be associated with decreased HF-related death rate in Caucasian-Brazilians.

Our study has some methodological limitations. First, the retrospective study design with regard to susceptibility of HF does not allow us to assess whether the *MMP-2* polymorphisms influence on LV remodeling and which alleles would be involved in this process. Second, our relatively small sample size did not allow to further explore the association of *MMP-2* polymorphisms with HF prognosis stratifying the analyses by potential modifiers such as age, ischemic/non-ischemic etiology, and duration of follow-up. For this same reason, the 3 haplotypes that had a frequency of ≥ 1% and carried at least 1 minor allele were grouped in 1 category and compared with the haplotype composed of the 3 major alleles (rather than analyzing each individual haplotype). Given the potential effect of gender on the development and progression of HF, we repeated the analyses stratifying by gender and ethnic group. In brief, the frequency distribution of *MMP-2* genotypes and haplotypes in HF patients and blood donors is in line with those observed for the joint analysis of males and females. Separate survival analysis for males and females showed similar profiles to those reported for males and females analyzed together. However, statistically significant differences between genotypes or haplotypes were detected only in Caucasian-Brazilian males, maybe because the number of males was almost twice than that of females (S2 Fig).

## Conclusions

Our study with a long-term follow-up does not exclude the possibility that polymorphisms in *MMP-2* gene might be associated with HF prognosis in Caucasian-Brazilians with reduced LVEF. However, given the lack of a replication cohort, the loss of association in the multivariate analyses, and the limitations considered above, our findings should be interpreted with caution and need to be addressed in further larger studies.

## Supporting Information

S1 Fig Survival analyses for all-cause death (column on the left) and HF-related death (column on the right) according to the -1575G>A, -1059G>A, and -790G>T polymorphisms in *MMP-2* in Caucasian- and African-Brazilians (Excluding Patients with Etiologies Other than Ischemic, Idiopathic, or Hypertensive HF). (DOC)

S2 Fig Survival analyses for all-cause death (column on the left) and HF-related death (column on the right) according to the -1575G>A, -1059G>A, and -790G>T polymorphisms in *MMP-2*, Stratified by Gender and Ethnic Group. (DOC)

S1 File Clinical Data of Heart Failure Patients. (XLS)

S1 Table Primers and Conditions of PCR-RFLP Method for Genotyping of *Matrix Metalloproteinase-2* Polymorphisms. (DOC)

S2 Table Genotype and Allele Frequencies of *Matrix Metalloproteinase-2* Gene Polymorphisms in Caucasian- and African-Brazilians. (DOC)

S3 Table Pairwise Linkage Disequilibrium between *Matrix Metalloproteinase-2* Gene Polymorphisms in Heart Failure Patients and Blood Donors. (DOC)

S4 Table Multivariate Analysis of the -790G>T Polymorphism for Heart Failure-Related Death in Caucasian-Brazilians. (DOC)

S5 Table Multivariate Analysis of *Matrix Metalloproteinase-2* Haplotypes for Heart Failure-Related Death in Caucasian-Brazilians. (DOC)

S6 Table Genotype and Allele Frequencies of *Matrix Metalloproteinase-2* Gene Polymorphisms in Caucasian- and African-Brazilians (Excluding Patients with Etiologies Other than Ischemic, Idiopathic, or Hypertensive HF). (DOC)
