# Supplementary material for: Matrix Metalloproteinase-2 Polymorphisms in Chronic Heart Failure: Relationship with Susceptibility and Long-Term Survival
Source: PLoS One. 2016 Aug 23;11(8):e0161666. doi: 10.1371/journal.pone.0161666 (PMC4995023; doi:10.1371/journal.pone.0161666)
Supplement: S2 Fig — (DOC) [file pone.0161666.s002.doc]

**
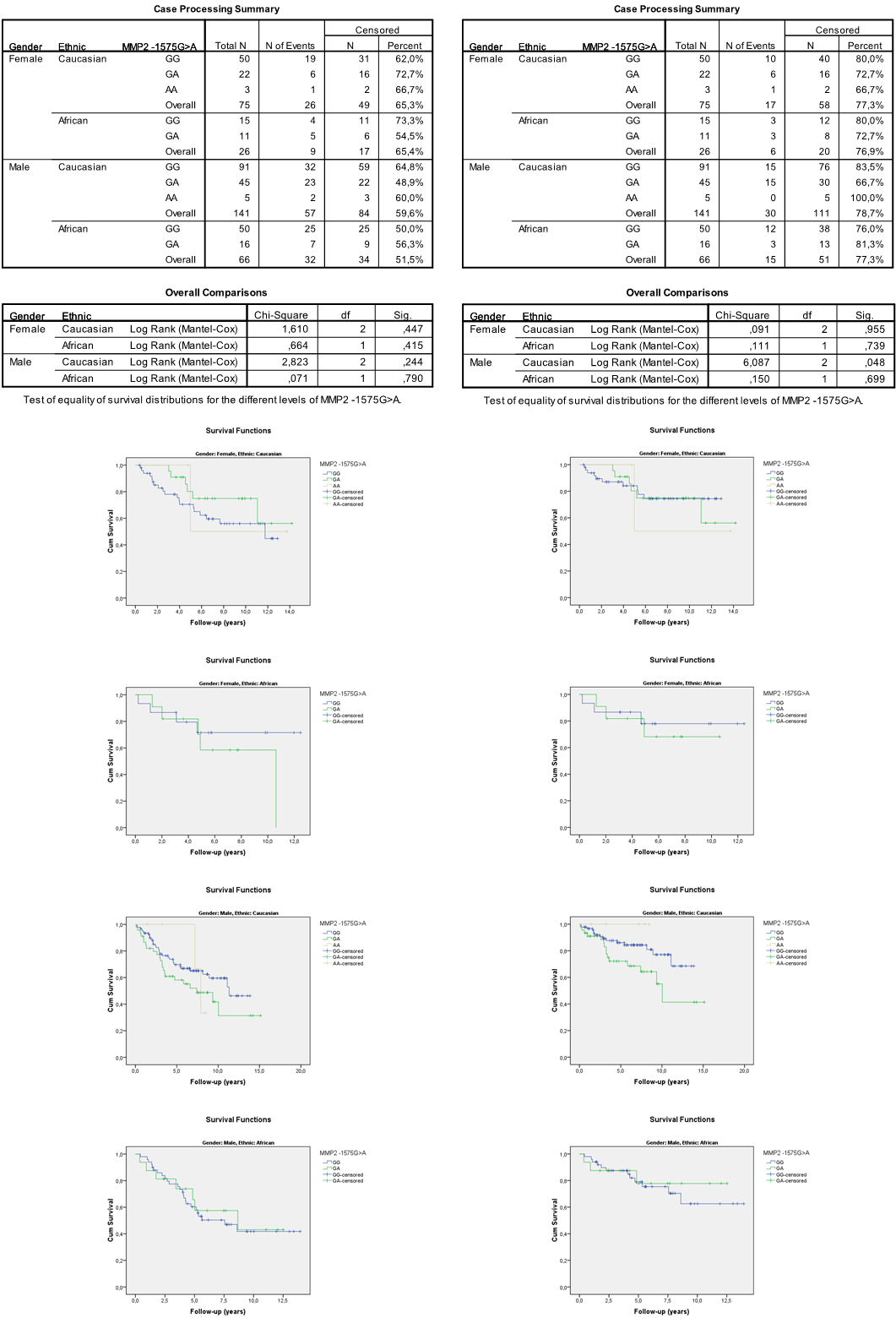
**

**
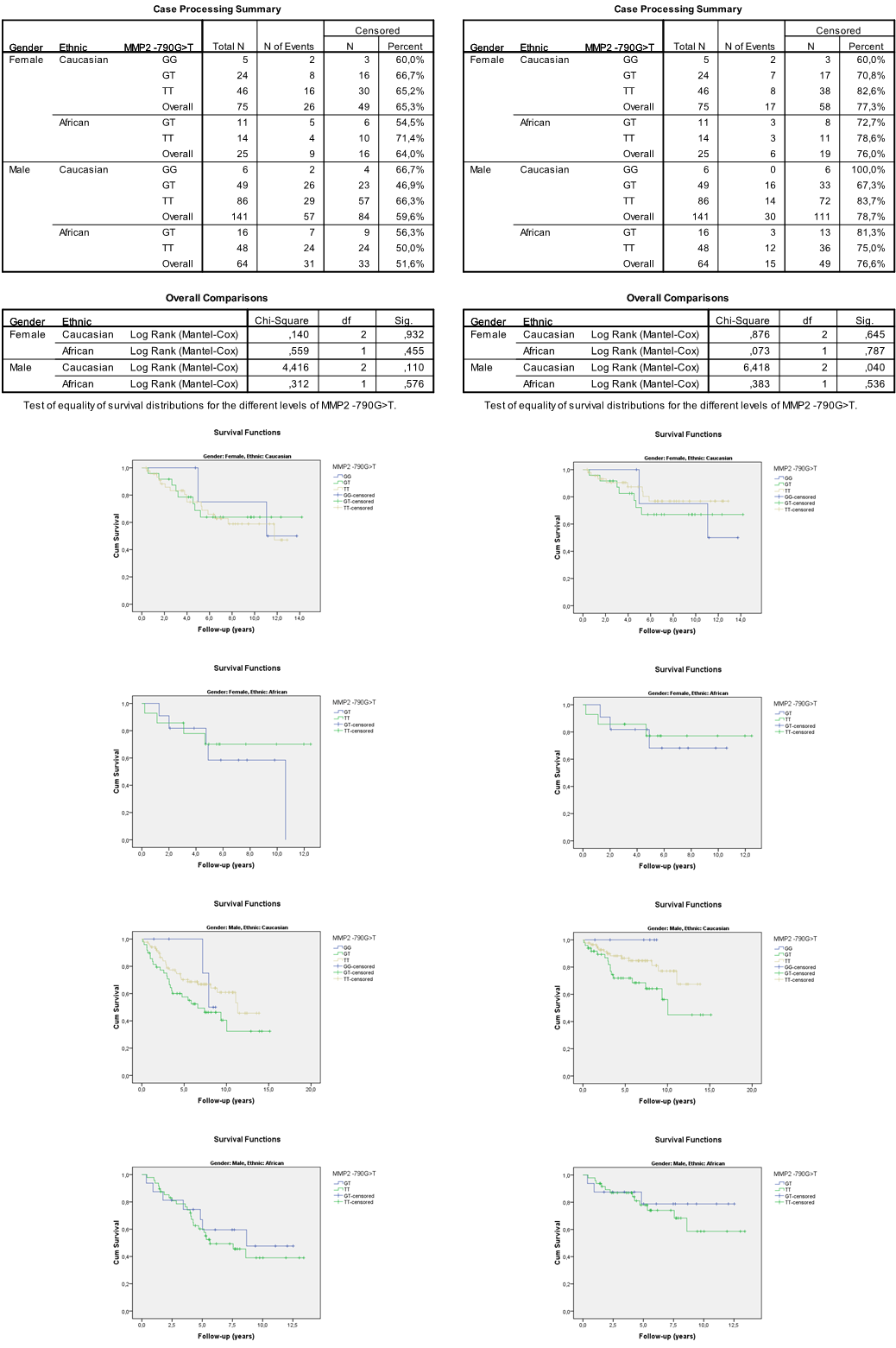
**

**
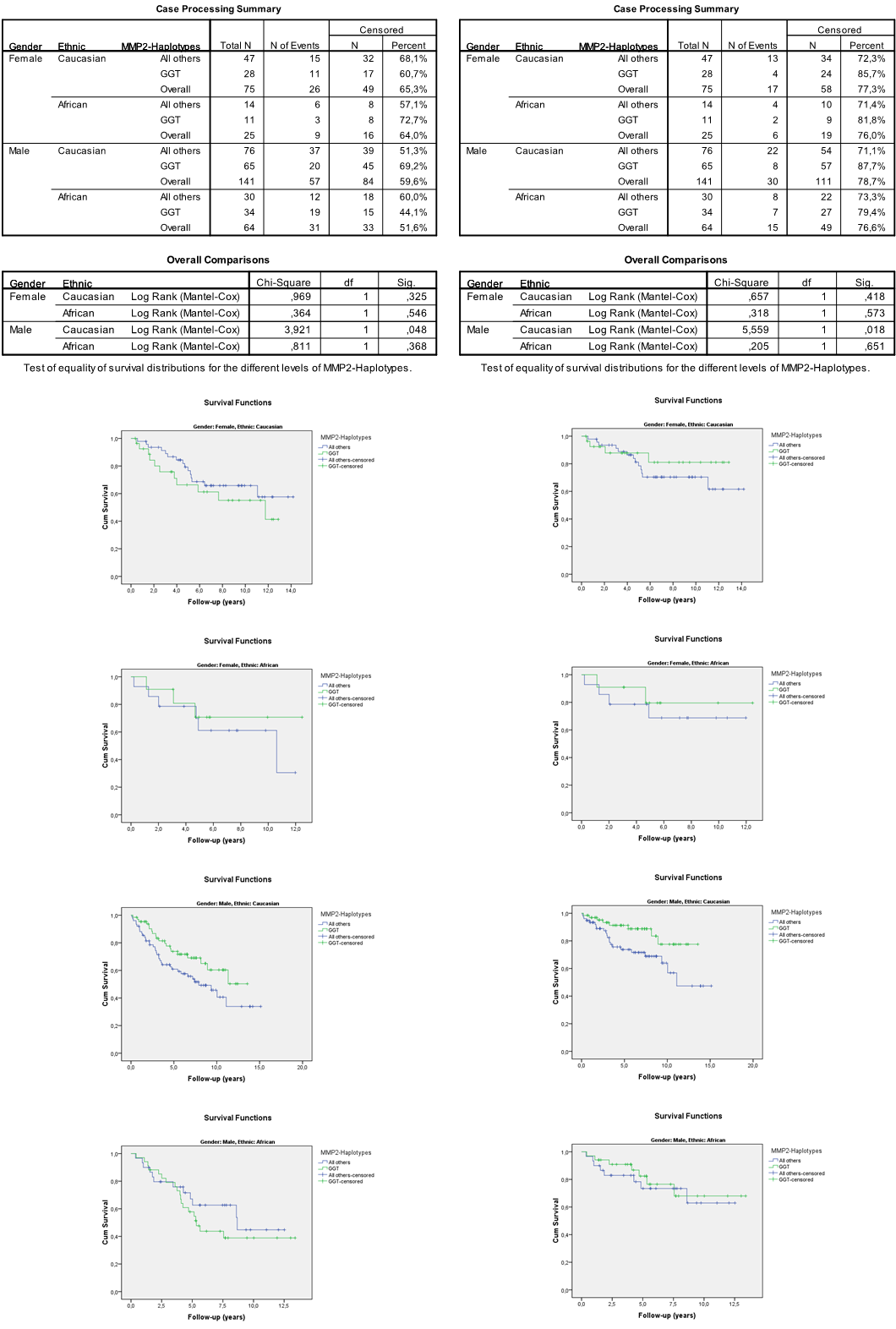
**

**S2 Figures. Survival analyses for all-cause death (column on the left) and HF-related death (column on the right) according to the -1575G>A, -1059G>A, and -790G>T polymorphisms in *MMP-2*, Stratified by Gender and Ethnic Group.**
