# Supplementary material for: Matrix Metalloproteinase-2 Polymorphisms in Chronic Heart Failure: Relationship with Susceptibility and Long-Term Survival
Source: PLoS One. 2016 Aug 23;11(8):e0161666. doi: 10.1371/journal.pone.0161666 (PMC4995023; doi:10.1371/journal.pone.0161666)
Supplement: S1 Table — (DOC) [file pone.0161666.s004.doc]

**Table S1. Primers and Conditions of PCR-RFLP Method for Genotyping of *Matrix Metalloproteinase-2* Polymorphisms.**

| **SNP** | **Sequence of primers^a^** | **Ta (°C)** | **Length of PCR product (bp)** | **Restriction enzyme** | **Restriction enzyme digest fragment (bp)** |
| --- | --- | --- | --- | --- | --- |
| -1575G>A  (rs243866) | 5’-AGAGCACACACCCACCAGACA-3’  5’-ACAGCAAGAGGCACTGGAGAAG-3’ | 65 | 301 | *BspH*I | G: 301  A: 206 + 95 |
| -1059G>A  (17859821) | 5’-CTCACCCTGTGCCCCACCTT-3’  5’-CATCCTCACTGCTCATTTTACGCTA-3’ | 65 | 554 | *Hha*I | A: 554  G: 374 + 180 |
| -790G>T  (rs243864) | 5’-CACTGGTGGGTGCTTCCTTTAAC-3’  5’-TGAGATAGAAATTGGGCAAGACTGGTTTACTA-3’ | 60 | 187 | *Dde*I | G: 187  T: 154 + 33 |

PCR, polymerase chain reaction; RFLP, restriction fragment length polymorphism; SNP, single nucleotide polymorphism; Ta, annealing temperature.

^a^ Described by Hua et al. [22].
