# Supplementary material for: Matrix Metalloproteinase-2 Polymorphisms in Chronic Heart Failure: Relationship with Susceptibility and Long-Term Survival
Source: PLoS One. 2016 Aug 23;11(8):e0161666. doi: 10.1371/journal.pone.0161666 (PMC4995023; doi:10.1371/journal.pone.0161666)
Supplement: S2 Table — (DOC) [file pone.0161666.s005.doc]

**Table S2. Genotype and Allele Frequencies of *Matrix Metalloproteinase-2* Gene Polymorphisms in Caucasian- and African-Brazilians.**

| **Polymorphisms** | **Heart Failure Patients** | | | | **Blood Donors** | | | |
| --- | --- | --- | --- | --- | --- | --- | --- | --- |
|  | **Caucasian- African- P^a^**  **Brazilians Brazilians** | | | | **Caucasian- African- P^a^**  **Brazilians Brazilians** | | | |
| -1575G>A |  | 216 | 92 |  | | 255 | 75 |  |
| Genotypes | GG | 141 (65.3) | 65 (70.7) | 0.154 | | **165 (64.7)** | **58 (77.4)** | **0.007** |
|  | GA | 67 (31.0) | 27 (29.3) |  | | **86 (33.7)** | **13 (17.3)** |  |
|  | AA | 8 (3.7) | 0 |  | | 4 (1.6) | 4 (5.3) |  |
| Alleles | G | 80.8 | 85.3 | 0.218 | | 81.6 | 86.0 | 0.256 |
|  | A | 19.2 | 14.7 |  | | 18.4 | 14.0 |  |
| -1059G>A |  | 206 | 86 |  | | 235 | 74 |  |
| Genotypes | GG | 157 (76.2) | 65 (75.6) | 0.481 | | 170 (72.4) | 55 (74.3) | 0.091 |
|  | GA | 47 (22.8) | 21 (24.4) |  | | 60 (25.5) | 14 (18.9) |  |
|  | AA | 2 (1.0) | 0 |  | | 5 (2.1) | 5 (6.8) |  |
| Alleles | G | 87.6 | 87.8 | > 0.999 | | 85.1 | 83.8 | 0.795 |
|  | A | 12.4 | 12.2 |  | | 14.9 | 16.2 |  |
| -790G>T |  | 216 | 89 |  | | 249 | 73 |  |
| Genotypes | GG | **11 (5.1)** | **0** | 0.064 | | 8 (3.2) | 4 (5.5) | **0.018** |
|  | GT | 73 (33.8) | 27 (30.3) |  | | **87 (34.9)** | **13 (17.8)** |  |
|  | TT | 132 (61.1) | 62 (69.7) |  | | **154 (61.9)** | **56 (76.7)** |  |
| Alleles | G | 22.0 | 15.2 | 0.071 | | 20.7 | 14.4 | 0.115 |
|  | T | 78.0 | 84.8 |  | | 79.3 | 85.6 |  |

Data are expressed as absolute number (percentage) or percentage.

^a^ P-values for the comparisons between Caucasian- and African-Brazilians were calculated using the Pearson chi-square or the likelihood-ratio chi-square test, as appropriate. Frequencies that deviate significantly from expected in the analysis of adjusted residuals and significant P-values are shown in bold.
