# Supplementary material for: Matrix Metalloproteinase-2 Polymorphisms in Chronic Heart Failure: Relationship with Susceptibility and Long-Term Survival
Source: PLoS One. 2016 Aug 23;11(8):e0161666. doi: 10.1371/journal.pone.0161666 (PMC4995023; doi:10.1371/journal.pone.0161666)
Supplement: S3 Table — (DOC) [file pone.0161666.s006.doc]

**Table S3. Pairwise Linkage Disequilibrium between *Matrix Metalloproteinase-2* Gene Polymorphisms in Heart Failure Patients and Blood Donors.**

|  | **Heart Failure Patients** | | | **Blood Donors** | | |
| --- | --- | --- | --- | --- | --- | --- |
|  | **-1575G>A** | **-1059G>A** | **-790G>T** | **-1575G>A** | **-1059G>A** | **-790G>T** |
| Caucasian-Brazilians | |  |  |  |  |  |
| -1575G>A | - | 0.033 | 0.792 | - | 0.039 | 0.864 |
| -1059G>A | -0.992 | - | 0.025 | -0.996 | - | 0.036 |
| -790G>T | 0.969 | -0.784 | - | 1.0 | -0.900 | - |
| African-Brazilians | |  |  |  |  |  |
| -1575G>A | - | 0.023 | 0.782 | - | 0.031 | 0.968 |
| -1059G>A | -0.989 | - | 0.023 | -0.996 | - | 0.032 |
| -790G>T | 0.902 | -0.962 | - | 1.0 | -0.996 | - |

D’ values form the lower left triangle and r^2^ values form the right upper triangle.
