# Supplementary material for: Matrix Metalloproteinase-2 Polymorphisms in Chronic Heart Failure: Relationship with Susceptibility and Long-Term Survival
Source: PLoS One. 2016 Aug 23;11(8):e0161666. doi: 10.1371/journal.pone.0161666 (PMC4995023; doi:10.1371/journal.pone.0161666)
Supplement: S4 Table — (DOC) [file pone.0161666.s007.doc]

**Table S4. Multivariate Analysis of the -790G>T Polymorphism for Heart Failure-Related Death in Caucasian-Brazilians.**

|  | **Hazard Ratio (95% CI)** | **P-value^a^** |
| --- | --- | --- |
| -790TT genotype (vs. GT) | 0.703 (0.365-1.353) | 0.291 |
| Cigarette smoking (pack-years) | 1.010 (1.002-1.018) | 0.012 |
| QRS duration (ms) | 1.011 (1.003-1.018) | 0.006 |
| Sodium (mEq/L) | 0.886 (0.798-0.984) | 0.024 |
| Hemoglobin (g/dL) | 0.746 (0.596-0.934) | 0.010 |

^a^ P-values were calculated using the Cox-proportional hazard model.
