# Supplementary material for: Matrix Metalloproteinase-2 Polymorphisms in Chronic Heart Failure: Relationship with Susceptibility and Long-Term Survival
Source: PLoS One. 2016 Aug 23;11(8):e0161666. doi: 10.1371/journal.pone.0161666 (PMC4995023; doi:10.1371/journal.pone.0161666)
Supplement: S5 Table — (DOC) [file pone.0161666.s008.doc]

**Table S5. Multivariate Analysis of *Matrix Metalloproteinase-2* Haplotypes for** Heart Failure-Related Death in Caucasian-Brazilians.

|  | **Hazard Ratio (95% CI)** | **P-value^b^** |
| --- | --- | --- |
| -1575G/-1059G/-790T homozygous haplotype^a^ | 0.521 (0.248-1.093) | 0.085 |
| Cigarette smoking (pack-years) | 1.011 (1.003-1.018) | 0.006 |
| QRS duration (ms) | 1.011 (1.004-1.019) | 0.003 |
| Sodium (mEq/L) | 0.899 (0.810-0.997) | 0.044 |
| Hemoglobin (g/dL) | 0.782 (0.634-0.963) | 0.021 |

^a^ Versus carriership of at least 1 copy of the -1575A/-1059G/-790G, -1575G/-1059G/-790G, or -1575G/-1059A/-790T haplotype. Only haplotypes with a frequency of ≥ 1% were included in this analysis.

^b^ P-values were calculated using the Cox-proportional hazard model.
