# Supplementary material for: Matrix Metalloproteinase-2 Polymorphisms in Chronic Heart Failure: Relationship with Susceptibility and Long-Term Survival
Source: PLoS One. 2016 Aug 23;11(8):e0161666. doi: 10.1371/journal.pone.0161666 (PMC4995023; doi:10.1371/journal.pone.0161666)
Supplement: S6 Table — (DOC) [file pone.0161666.s009.doc]

**Table S6. Genotype and Allele Frequencies of *Matrix Metalloproteinase-2* Gene Polymorphisms in Caucasian- and African-Brazilians (Excluding Patients with Etiologies Other than Ischemic, Idiopathic, or Hypertensive HF).**

| **Polymorphisms** |  | **Caucasian-Brazilians** | | | **African-Brazilians** | | |
| --- | --- | --- | --- | --- | --- | --- | --- |
|  |  | **Heart Failure^a^** | **Blood Donor** | **P-value^b^** | **Heart Failure^a^** | **Blood Donor** | **P-value^b^** |
| -1575G>A |  | 193 | 255 |  | 77 | 75 |  |
| Genotypes | GG | 128 (66.3) | 165 (64.7) | 0.301 | 56 (72.7) | 58 (77.4) | **0.024** |
|  | GA | 58 (30.1) | 86 (33.7) |  | 21 (27.3) | 13 (17.3) |  |
|  | AA | 7 (3.6) | 4 (1.6) |  | **0** | **4 (5.3)** |  |
| Alleles | G | 81.3 | 81.6 | > 0.999 | 86.4 | 86.0 | > 0.999 |
|  | A | 18.7 | 18.4 |  | 13.6 | 14.0 |  |
| -1059G>A |  | 183 | 235 |  | 71 | 74 |  |
| Genotypes | GG | 140 (76.5) | 170 (72.4) | 0.510 | 51 (71.8) | 55 (74.3) | **0.018** |
|  | GA | 41 (22.4) | 60 (25.5) |  | 20 (28.2) | 14 (18.9) |  |
|  | AA | 2 (1.1) | 5 (2.1) |  | **0** | **5 (6.8)** |  |
| Alleles | G | 87.7 | 85.1 | 0.327 | 85.9 | 83.8 | 0.732 |
|  | A | 12.3 | 14.9 |  | 14.1 | 16.2 |  |
| -790G>T |  | 193 | 249 |  | 74 | 73 |  |
| Genotypes | GG | 10 (5.2) | 8 (3.2) | 0.567 | **0** | **4 (5.5)** | **0.018** |
|  | GT | 64 (33.2) | 87 (34.9) |  | 22 (29.7) | 13 (17.8) |  |
|  | TT | 119 (61.6) | 154 (61.9) |  | 52 (70.3) | 56 (76.7) |  |
| Alleles | G | 21.8 | 20.7 | 0.759 | 14.9 | 14.4 | > 0.999 |
|  | T | 78.2 | 79.3 |  | 85.1 | 85.6 |  |

| **Polymorphisms** |  | **Caucasian-Brazilians** | | | **African-Brazilians** | | |
| --- | --- | --- | --- | --- | --- | --- | --- |
|  |  | **Heart Failure^a^** | **Blood Donor** | **P-value** | **Heart Failure^a^** | **Blood Donor** | **P-value** |
| Haplotype |  | 386 | 504 |  | 148 | 146 |  |
|  | GGT | 0.6604 | 0.6470 | 0.688 | 0.7028 | 0.6894 | 0.248 |
|  | GGG | 0.0297 | 0.0192 |  | 0.0197 | < 0.0001 |  |
|  | GAT | 0.1167 | 0.1441 |  | 0.1418 | 0.1668 |  |
|  | GAG | 0.0066 | 0.0034 |  | 0.0006 | - |  |
|  | AGT | 0.0052 | 0.0021 |  | 0.0068 | < 0.0001 |  |
|  | AGG | 0.1812 | 0.1841 |  | 0.1281 | 0.1438 |  |
|  | AAT | 0.0001 | < 0.0001 |  | - | - |  |
|  | AAG | 0.0001 | 0.0001 |  | 0.0002 | < 0.0001 |  |

^a^ Ischemic, idiopathic or hypertensive HF (excluding all other etiologies).

Data are expressed as absolute number (percentage), percentage or relative frequency.

^b^ P-values for the comparisons between Caucasian- and African-Brazilians were calculated using the Pearson chi-square or the likelihood-ratio chi-square test, as appropriate. Frequencies that deviate significantly from expected in the analysis of adjusted residuals and significant P-values are shown in bold.

Haplotypes are shown in the following chromosomal order: -1575G>A, -1059G>A, and -790G>T. Haplotype frequencies were estimated and compared among the groups by a permutation test in PHASE software. Relative haplotypes frequencies are based on the total number of chromosomes (instead of number of subjects).
